# Comparison of Regional Cerebral Blood Flow Responses to Hypoglycemia Using Pulsed Arterial Spin Labeling and Positron Emission Tomography

**DOI:** 10.1371/journal.pone.0060085

**Published:** 2013-03-28

**Authors:** Ana Maria Arbeláez, Yi Su, Jewell B. Thomas, Amy C. Hauch, Tamara Hershey, Beau M. Ances

**Affiliations:** 1 Department of Pediatrics, Washington University School of Medicine, St. Louis, Missouri, United States of America; 2 Department of Radiology, Washington University School of Medicine, St. Louis, Missouri, United States of America; 3 Department of Neurology, Washington University School of Medicine, St. Louis, Missouri, United States of America; 4 Department of Psychiatry, Washington University School of Medicine, St. Louis, Missouri, United States of America; University of Minnesota, United States of America

## Abstract

Different brain regions sense and modulate the counterregulatory responses that can occur in response to declining plasma glucose levels. The aim of this study was to determine if changes in regional cerebral blood flow (rCBF) during hypoglycemia relative to euglycemia are similar for two imaging modalities–pulsed arterial spin labeling magnetic resonance imaging (PASL-MRI) and positron emission tomography (PET). Nine healthy non-diabetic participants underwent a hyperinsulinemic euglycemic (92±3 mg/dL) – hypoglycemic (53±1 mg/dL) clamp. Counterregulatory hormone levels were collected at each of these glycemic levels and rCBF measurements within the previously described network of hypoglycemia-responsive regions (thalamus, medial prefrontal cortex and globus pallidum) were obtained using PASL-MRI and [^15^O] water PET. In response to hypoglycemia, rCBF was significantly increased in the thalamus, medial prefrontal cortex, and globus pallidum compared to euglycemia for both PASL-MRI and PET methodologies. Both imaging techniques found similar increases in rCBF in the thalamus, medial prefrontal cortex, and globus pallidum in response to hypoglycemia. These brain regions may be involved in the physiologic and symptom responses to hypoglycemia. Compared to PET, PASL-MRI may provide a less invasive, less expensive method for assessing changes in rCBF during hypoglycemia without radiation exposure.

## Introduction

Hypoglycemia is a devastating problem for people with diabetes and accounts for 6–10% of all deaths of people with Type 1 diabetes [1]. Recurrent hypoglycemia in diabetes occurs due to the interplay between therapeutic hyperinsulinemia and compromised counterregulatory and symptom responses to falling plasma glucose levels. This can lead to hypoglycemia associated autonomic failure (HAAF) and can cause a vicious cycle of repeated hypoglycemia [2], [3]. The exact mechanisms involved in the normal physiologic responses to hypoglycemia as well as their impairment in HAAF are not well known. A group of brain regions with glucose-sensing cells has been hypothesized to be involved [4]. These cells are unique in that they are able to translate a fall in extracellular glucose into a change in neurotransmitter or hormone release [5]. Thus, better delineation of this hypoglycemic network and how it is integrated into the hypoglycemic responses is key in order to develop novel therapeutic interventions that prevent recurrent hypoglycemia or improve hypoglycemia awareness in type 1 diabetes.

Changes in regional cerebral blood flow (rCBF) in response to hypoglycemia have been assessed using [^15^O] water positron emission tomography (PET) [4], [6], [7], [8]. Regional increases in rCBF may reflect increases in neuronal activity due to rises in synaptic metabolic activity but not spike firing rate [9], [10], [11]. Observed increases in synaptic activity in response to hypoglycemia, have been reported using PET within a discrete network of interconnected brain regions that included the medial prefrontal cortex, orbitoprefrontal cortex, globus pallidus, thalamus, and periaqueductal grey matter [4]. These same regions are involved in modulating visceral responses [12]. In addition, increased rCBF has been observed in the dorsal midline thalamus during recurrent hypoglycemia, accompanied by attenuated counterregulatory responses due to HAAF [6]. While hypoglycemic changes in rCBF have primarily been assessed by PET, this imaging technique has significant limitations including the need for a cyclotron, significant expense, and the injection of a radiolabeled tracer thus limiting the number of studies that can be performed in a particular subject and restricting studies to adults only. More recently, non-invasive MRI methods have been developed to assess rCBF [13] and provide comparable CBF quantification between PET and ASL; at least in euglycemia [14].

In the pulsed arterial spin labeling magnetic resonance imaging (PASL-MRI) method, arterial blood water is magnetically labeled just proximal to the region (slice) of interest. Water molecules within this portion of arterial blood are labeled magnetically. This ‘paramagnetic tracer’ flows into a slice of interest and exchanges with tissue water. The inflowing blood water alters tissue magnetization. During this time, the “tag” image is obtained. This process is then repeated without labeling arterial blood to create the “control” image. The difference between the “control” and “tag” image produces a perfusion image which reflects the amount of arterial blood delivered to each voxel within the slice within the transit time. PASL-MRI provides reproducible and reliable quantitative CBF measurements across the brain. Compared to PET, PASL-MRI is completely noninvasive, less expensive, has no radiation exposure risk, is performed without gadolinium, thus bypassing concerns regarding nephrogenic systemic fibrosis in patients with significant renal insufficiency and can be repeatedly performed on the same subject and on subjects of any age with great ease [15]. However, this technique has been used in only a limited number of clinical applications (i.e. brain tumors, Alzheimer’s Disease or stroke) [16], [17], [18] and has not been widely used to study hypoglycemia or HAAF [19], [20].

The aim of this study was to determine, using a within subjects study design, if PASL-MRI can detect a pattern of changes in rCBF due to hypoglycemia similar to that previously-observed with PET.

## Research Design and Methods

### Ethics Statement

The study was approved by the Washington University in Saint Louis (WUSTL) Human Research Protection Office. Each participant gave his or her written consent to participate in this study. All clinical investigation was conducted according to the principles expressed in the Declaration of Helsinki.

### Subjects

Nine healthy adult individuals (4 women and 5 men) who were mean ±SE age 29.7±2.6 years and mean body mass index (BMI) 24.2±1.1 kg/m^2^ were recruited through volunteers for health program at (WUSTL). All subjects were right handed and in good health, based on a medical history and physical examination. None were taking medications (aside from an oral contraceptive) that could affect rCBF or counterregulation. All had normal fasting plasma glucose, creatinine and hematocrit. None of the individuals had a personal or family history of diabetes in first degree relatives. None had a personal history of significant psychiatric, neurological, or cardiovascular conditions.

### Experimental Design

**Figure 1:**
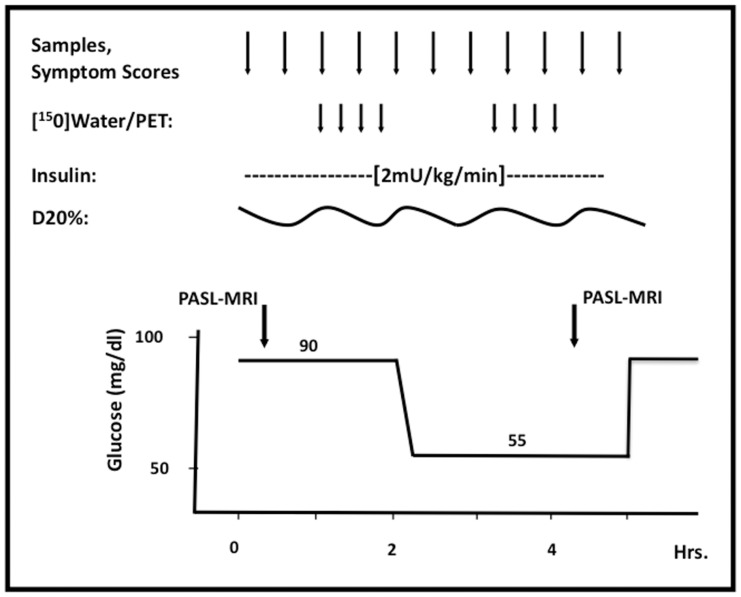
Schematic diagram of experimental protocol. Counterregulatory hormone levels, hypoglycemic symptom scores and rCBF measurements were obtained during the hyperinsulinemic euglycemic – hypoglycemic clamp. rCBF measurements were obtained using PASL-MRI and [^15^O]water PET for healthy individuals (*n* = 9).

Participants were studied in the morning after a 10-hour overnight fast. They remained supine throughout the study. Two intravenous catheters were inserted into arm veins (one for infusion of insulin and dextrose and the other for injection of radioactive isotope) and one intravenous catheter was inserted into a dorsal hand vein that was kept in a ∼55°C plexiglass box (for arterialized venous sampling). All nine subjects underwent a hyperinsulinemic (regular human insulin, Novo Nordisk, Bagsværd, Denmark in a dose of 2.0 mU·kg−1·min.−1) euglycemic (92±3 mg/dL [5.1 mmol/L] ∼2 hours) and then hypoglycemic (53±1 mg/dL [3.0 mmol/L]) x∼2 hours) clamp procedure using variable infusions of 20% dextrose based on plasma glucose determinations (YSI Glucose Analyzer 2, Yellow Springs Instruments, Yellow Springs, OH) every five minutes (Figure 1). During the euglycemic period, two PASL-MRI measurements of rCBF were obtained. In the second hour of euglycemia, [^15^O]water PET measurements were performed four times at 15 minute intervals. Hypoglycemia was then induced and PET measurements were obtained four times at 15 minute intervals. Two PASL-MRI measurements were obtained at the end of the hypoglycemic clamp. Each participant was moved between the PET and MRI scanners quickly while maintaining hypoglycemic and euglycemic conditions. All scans were obtained after the subject had reached at least 20 minutes of steady state at the desired glycemic level. Every 30 minutes, arterialized venous samples were drawn for analytes (glucose, insulin, glucagon, epinephrine, norepinephrine) and blood pressures and heart rates were recorded. Throughout the study cardiac function was monitored using an electrocardiogram.

The severity of the hypoglycemic symptoms was determined every 30 minutes during the euglycemic - hypoglycemic clamp with a validated questionnaire [21]. Subjects were asked to score (from 0, none, to 6, severe) six neurogenic (autonomic) symptoms (heart pounding, shaky/tremulousness and nervous/anxious (adrenergic) and sweaty, hungry and tingling (cholinergic)) and six neuroglycopenic symptoms (difficulty thinking/confused, tired/drowsy, weak, warm, faint and dizzy) [21].

### Analyte Measurements

Plasma glucose concentrations were measured with a glucose oxidase method (YSI Glucose Analyzer, Yellow Springs Instruments, Yellow Springs, OH). Plasma insulin concentrations were measured with a two site chemiluminescent assay (Immulite 1000, Siemens Corp, Los Angeles, CA). Counterregulatory hormones including plasma epinephrine and norepinephrine were measured with a single isotope derivative (radioenzymatic) method [22] and plasma glucagon concentrations were measured with a radioimmunoassay (Millipore, Temecula, CA).

### Magnetic Resonance (MR) Imaging Acquisition

All MRI imaging was performed on a 1.5T Siemens Avanto System (Erlangen, Germany) with a 12 channel radiofrequency head coil. To enable computation of rCBF, a total of four proximal inversion with control for off-resonance effects (PICORE Q2) PASL-MRI perfusion scans (3 minutes in duration) were collected with two at the initiation of euglycemia and two more at the end of the hypoglycemic clamp. Scanning parameters were TR = 2500 ms, TE = 13.0 ms, TI_1_ = 700 ms, TI_2_ = 1800 ms, flip angle = 90°, 100 mm tag with a 10 mm gap between the tag and the imaging slice, FOV = 256 mm, 4.0×4.0×8.0 mm voxels. A total of 140 PASL-MRI frames (or 70 PASL-MRI pairs) were analyzed for each subject. One high-resolution Tı-weighted magnetization-prepared rapid gradient echo (MPRAGE) [TR = 2400.0 ms, TE = 3.23 ms TI = 1000.0 ms, flip angle = 8°, 1×1×1 mm voxels] structural scan was also obtained for anatomical region definition and facilitate image alignment.

### PET Scan Acquisition

General details of the [^15^O]water PET acquisition and analysis methods have been previously reported in detail in prior publications [6]. All PET images were acquired with a Siemens/CTI (Knoxville, TN) EXACT HR+962 tomograph using the two-dimensional mode (interslice septa extended). Subjects were positioned in the scanner so that the entire brainstem was included within the 15 cm axial field of view. A transmission scan was collected at each scan session for PET data reconstruction. Four boluses of 50 mCi of [^15^O]water (1.85 GBq) were injected at 15 minute intervals [23], [24], [25] during euglycemia and repeated during hypoglycemia and 40-second emission scans were collected to measure relative rCBF.

### MR Image Post-Processing

In order to evaluate the PASL-MRI data, we developed a suite of in-house image processing utilities following previously established best practices [26]. The two PASL-MRI image series for each subject were cross-aligned and motion-corrected according to a rigid body algorithm using a program developed in-house [27]. “Tag” and “control” images were pairwise subtracted to obtain mean perfusion images. CBF was calculated for each mean perfusion image using the following formula [28], [29].
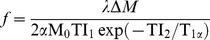
Where ΔM is a perfusion image calculated by pairwise subtraction of label and control images, M_0_ represents the image intensity of brain tissue at magnetic equilibrium, α represents the efficiency of tag delivery, λ is blood/tissue water partition coefficient, T_1a_ is the longitudinal relaxation time of blood, and TI_1_ and TI_2_ are the time required to deliver the tag and the time required to collect the image, respectively. The following values were used for this study: α = 0.98, λ = 0.9 mL/g, T_1a_ = 1.6 s [29].

**Figure 2:**
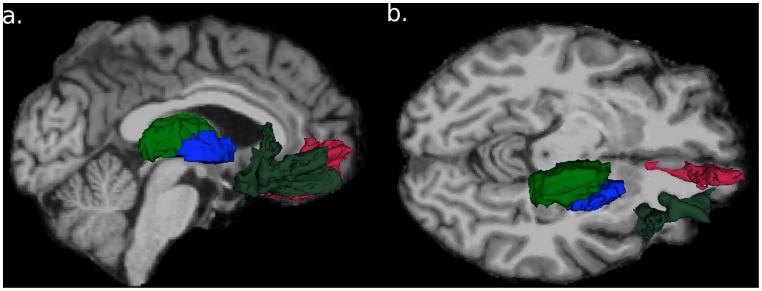
Free Surfer regions of interest. Free Surfer maps of ROI used for this analysis. A) Sagittal (top) B) Axial (bottom) view of Freesurfer ROIs. Blue: globus pallidum, Green: caudate, Dark Green: lateral prefrontal cortex, Magenta: medial prefrontal cortex.

In order to use PASL-MRI and PET methodology to assess if regional changes in rCBF occur in a-priori regions previously reported to have increased synaptic activity during hypoglycemia, each subject’s MPRAGE was segmented using FreeSurfer’s segmentation and cortical reconstruction analysis software package (Freesurfer Version 5.1 developed at the Martinos Center, Harvard University, Boston, MA; http://surfer.nmr.mgh.harvard.edu) [30], [31], [32]. Our regional analysis primarily focused on a network of brain areas [including the thalamus (thalamus proper), medial prefrontal cortex (rostral anterior cingulate), right orbital prefrontal cortex and globus pallidum] (Figure 2) that have been shown to have increased blood flow during hypoglycemia with PET [4]. Mean rCBF of each of these regions was obtained for both glycemic conditions.

### PET Scan Analyses

PET images were reconstructed using filtered back-projection technique [33]. All individual PET images were co-registered using standard technique [34] and normalized to a whole brain CBF of 50 mL/100 g/min. For each participant, the PET scans were registered to corresponding MPRAGE images using a vector-gradient based method [35]. Images from each glycemic conditions for individual subject were averaged to increase statistical precision [36]. The same regional analysis approach as the PASL-MRI data was used for PET CBF analysis.

### Voxel-wise Analysis of rCBF in PET and PASL-MRI

To allow between-subject voxel-wise comparison of PET and PASL-MRI, each subject’s MPRAGE data were warped to the 1988 Talairach atlas [37]. In order to eliminate anatomical differences as a possible confound in our analysis, a common atlas space was computed through an affine cross-registration of the Talairach-conformed structural scans from each individual subject [38]. For this analysis, the second PASL-MRI scan was aligned to the first PASL-MRI scan. The PASL-MRI scans were aligned to a study-specific derived atlas. Each computed rCBF map (whether PET or PASL-MRI) was transformed to this study-specific atlas space after rCBF computation [26]. A voxel-wise comparison of rCBF for the two conditions (euglycemia vs. hypoglycemia) was carried out separately for PET and PASL-MRI.

### Statistical Analysis

rCBF and relevant systemic variables were compared across conditions (during euglycemia vs. during hypoglycemia) using a paired t-test. P values less than 0.05 were considered significant. Hypoglycemia and euglycemia rCBF measures were expressed as means and SE. Coefficient of variation (COV) for each imaging method was calculated by computing the average SD of the rCBF from a particular region of interest (ROI) and dividing it by the mean of the rCBF from that area.

## Results

### Counterregulatory Hormone and Symptom Responses

During the hypoglycemic clamp plasma epinephrine responses (30±4 vs 348±37 pg/mL, p<0.0001), plasma glucagon (40±3 vs 91±3 pg/mL, p = 0.001), and cortisol (10±1 vs 21±2 µg/dL, p = 0.001) levels were significantly increased compared to euglycemia. Insulin levels during euglycemia were 160±4 vs 159±5 during hypoglycemia (p = 0.6). Hypoglycemic symptom scores significantly increased during hypoglycemia (2±0.4 vs 9±2, p = 0.005) (Table 1).

**Table 1:** Mean (±SE) plasma glucose and counterregulatory hormones at baseline and at time of CBF acquisition for euglycemic and hypoglycemic clamp phases and peak levels during each phase.

	EUGLYCEMIA			HYPOGLYCEMIA		
	Mean at Baseline	Mean during CBF acquisition	Peak value	Mean at Baseline	Mean during CBF acquisition	Peak value
**Glucose (mg/dl)**	91±1	92±3	101	54±1	53±1	58
**Insulin**	10±1	160±4	244	158±16	159±5	238
**Epinephrine (pg/ml)**	25±8	30±4	95	353±26	348±37	517
**Glucagon (pg/ml)**	59±7	40±3	62	85±11	91±3	169
**Cortisol (mg/dl)**	9±1	10±1	17	17±2	21±2	42
**Symptoms**	1±0.3	2±0.4	4	9±2	9±2	22

### Regional Cerebral Blood Flow

**Figure 3:**
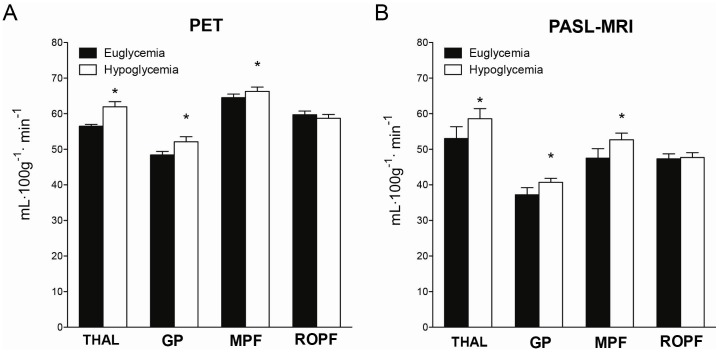
Qualitative cerebral blood flow during hypoglycemia and euglycemia in PET and PASL-MRI. Axial images showing the mean difference (CBF hypoglycemia – CBF euglycemia) maps from nine subjects for A) positron emission tomography (PET) and B) pulse arterial spin labeling magnetic resonance imaging (PASL-MRI). Yellow/orange represents increased and blue represents decreased blood flow during the hypoglycemic relative to euglycemic session. Similar increases in CBF for hypoglycemia were seen for both methods within the thalamus.

**Figure 4:**
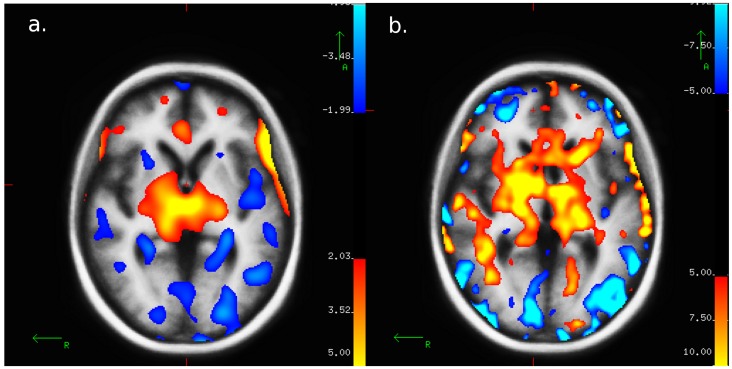
Quantitative regional cerebral blood flow during euglycemia and hypoglycemia in PET and PASL-MRI. Quantitative regional CBF (mL^.^100 g^−1.^ min^−1^) responses for euglycemia (black bars) and hypoglycemia (white bars) within a previously identified network. Significant increases in CBF were seen with hypoglycemia within the thalamus (THAL), globus pallidum (GP), and medial prefrontal cortex (MPF) for both A) PET and B) PASL-MRI. No significant increases were seen in the right orbitoprefrontal cortex (ROPF). *p<0.05.

During hypoglycemia, rCBF significantly increased within many ROIs for both PET and PASL-MRI (Figure 3). A significant increase in rCBF was confirmed in the thalamus (p = 0.003, p = 0.02), medial prefrontal cortex (p = 0.03, p = 0.01) and globus pallidum (p = 0.001, p = 0.01) during hypoglycemia compared to euglycemia for both PET and PASL-MRI respectively (Figure 4). The coefficient of variation across the ROI for PET was 6% and 12% for PASL-MRI, which are comparable between the two methods.

A Voxel-wise analysis of the brain using FreeSurfer’s general linear model tool was utilized to assess if certain brain regions were significantly increased for hypoglycemia for both methods. The only region that survived a multiple comparison analysis by both PASL-MRI and PET was the thalamus.

## Discussion

A fall in blood glucose level below ∼80 mg/dL induces a rapid counterregulatory response to restore euglycemia. This process involves the activation of peripheral and central glucose-sensing units involved in the physiological control of counterregulation. The mechanisms by which some patients with diabetes develop impaired responses to hypoglycemia remain unclear. A better understanding of these changes and the brain regions involved in the central integration of these responses, might allow targeted therapies aimed at preventing the loss of these responses in HAAF, and allow diabetic patients safer glycemic control. Our findings demonstrate that acute hypoglycemia activates the thalamus, the medial prefrontal cortex and the globus pallidum, as has previously been shown in studies using [^15^O]water PET [4]. These regions mediate autonomic responses to various visceral stimuli and may facilitate mechanisms due to hypoglycemia [39], [40], [41], [42]. Although our study does not allow us to examine whether the changes seen in rCBF lead to the specific hormonal changes, based on prior studies, the thalamus has connections to the hypothalamus that can regulate the sympathoadrenal outflow during hypoglycemia, and its activation seem to be independent of awareness and be involved primarily with counterregulatory responses to hypoglycemia [7]. Meanwhile, the medial prefrontal cortex seems to be involved in counterregulation [8] and symptomatic awareness of hypoglycemia, and its activation during acute hypoglycemia may be caused by hypoglycemia per se, rather than by counterregulation [43]. Further studies are needed to outline the hierarchy of activation of brain regions and its association with hypoglycemic physiological and behavioral responses.

That we only found significant increases in rCBF in the thalamus using a voxel wise approach by both ASL and PET, does not mean that the increases in rCBF in the other regions found in the regional analysis are incorrect. A voxel-wise whole brain analysis is less sensitive in detecting changes in *a priori* identified regions due to correction for multiple comparisons and a factor of multiple thresholding across the entire brain space.

Although PASL-MRI has been already used in hypoglycemia studies [19], [20], this study documents that PASL-MRI is a reliable method that can be used to study activation of the hypoglycemia network in a noninvasive manner since our work is the first to compare this technique to the gold standard in both conditions. PASL-MRI has several advantages, including that it is a less invasive and expensive method compared to PET, it does not require placement of an arterial line or any radiation exposure, and the scanning time can be shorter so that successive rCBF maps using PASL-MRI can be acquired on the same subject. These significant advantages encourage extension of this noninvasive imaging methodology to future pediatric studies (where less invasive methodologies are preferred) as well as to other studies of healthy and diabetic individuals, which seek to study hypoglycemia-associated recruitment of brain regions and other hypoglycemia-associated physiological responses more broadly. Being able to study children, particularly diabetic children, offers better opportunities to understand the early loss of counter-regulatory response in recurrent hypoglycemia by allowing us to follow the full course of diabetes. However, limitations exist for PASL-MRI. It can only be performed on patients that can tolerate MRI and have no braces or metal implants that wouldn’t limit the implementation of this technique. This technique also requires a very high signal-to noise ratio and cannot accurately map either low (<10 mL/100 g/minute) or high (>150 mL/100 g/minute) rCBF states [13].

The most important finding of our work is the consistent increase in rCBF due to hypoglycemia in the same regions across both imaging methods. Our work suggests that there is presence of a cerebral network involved in the hypoglycemic responses and demonstrates the ability to use this novel MRI method in a reliable manner to study these responses.
